# Usefulness of Pyloromyotomy With Transhiatal Esophagectomy in Improving Gastric Emptying

**DOI:** 10.4021/gr346w

**Published:** 2011-09-20

**Authors:** Rahim Mahmodlou, Nazmohammad Badpa, Emad Nosair, Hojat Shafipour, Mohammad Ghasemi-rad

**Affiliations:** a Department of Thoracic Surgery, Urmia University of Medical Sciences, Urmia, Iran; b Department of Surgery, Urmia University of Medical Sciences, Urmia, Iran; c Department of Anatomy, Sharjeh University of Medical Sciences, United Arab Emirates; d Department of Nuclear Medicine, Urmia University of Medical Sciences, Urmia, Iran; e Student Research Center (SRC), Urmia University of Medical Sciences, Urmia, Iran

**Keywords:** Pyloromyotomy, Transhiatal esophagectomy, Gastric emptying

## Abstract

**Introduction:**

Pyloromyotomy is a pyloric drainage procedure routinely done during transhiatal esophagectomy (THE) to prevent delayed gastric emptying (GE) resulting from truncal vagotomy. However, controversy still surrounds the need for pyloric drainage following esophageal substitution with gastric conduit after esophagectomy. The aim of this study was to determine the usefulness of pyloromyotomy in improving the postoperative gastric emptying time.

**Methods:**

Forty patients with esophageal cancer underwent THE. 20 patients underwent THE without pyloromyotomy (group A), while the other 20 patients (group B) underwent THE with pyloromyotomy. Using Technetium-99 m, gastric scintigraphy-using gamma camera, was done for all the patients 6 months post-surgery to measure the gastric half emptying time (T50).

**Results:**

For the liquid phase, the mean (T50) in the patients without pyloromytomy (group A) was 74.5 ± 56.71 minutes ± SD versus 62.85 ± 59.35 minutes ± SD in the patients with pyloromytomy (group B) which is not significant (P = 0.529). For the solid phase, the mean (T50) in patients of group A was 139.40 ± 94.156 minutes ± SD versus 141.15 ± 48.423 minutes ± SD in group B (P value 0.941) which is also not significant.

**Conclusion:**

Six months after THE, pyloromyotomy done with THE showed no significant value on affecting the mean gastric emptying time compared to those underwent THE without pyloromyotomy.

## Introduction

It has been reported during the past two decades that transhiatal esophagectomy (THE) is a relatively safe and well-tolerated operation for most patients requiring esophageal resection for benign and malignant diseases. It has relatively fewer complications and less morbidity than the traditional transthoracic approaches if performed with care and for the proper indications [1-3]. While replacing the excised part of esophagus with gastric conduit, truncal vagotomy is performed; and routinely a pyloric drainage procedure might follow.

However, controversy still surrounds the need for pyloric drainage following esophageal substitution with gastric conduit after esophagectomy. Although trials have addressed the need for pyloric drainage after esophageal substitution, the variables of surgical method, choice of conduit (whole stomach or gastric tube), conduit position, and the anastomotic location confound the analysis. Pyloric drainage reduces the incidence of aspiration pneumonia and improves early postoperative outcome. It also reduces the occurrence of early gastric outlet obstruction (GOO), but it had little effect on mortality, pulmonary morbidity and late postoperative foregut function [4].

On the other hand, previous studies documented the uselessness of pyloric drainage procedures by either pyloroplasty or pyloromyotomy following esophageal substitution with gastric conduit. Only a minority of patients develops GOO after esophagectomy, and they may predispose to dumping and duodenal bile reflux, and thus impairing late postoperative functional outcome [5]. They may interfere with mobilization of the stomach by shortening or anchoring the gastric outlet [6]. Moreover, it has been reported that gastric drainage following esophagectomy has no influence on the delayed gastric emptying (GE) [7], and the foregut function improves with time, regardless of a pyloric drainage procedure [8, 9].

The need to perform a drainage procedure after esophagectomy is historically derived from experience with truncal vagotomy for peptic ulcer disease [10]. It became apparent that pyloroplasty or pyloromyotomy could potentially alleviate the emptying delay associated with a vagotomized stomach.

Establishing pyloric drainage after esophagectomy with complete vagotomy has not been widely accepted as standard of therapy in high volume esophageal centers. Despite evidence to support gastric drainage procedures following esophagectomy, there is an equivalent body of evidence that attests to the adequacy of spontaneous emptying with an intact pylorus. Interestingly, there are data to suggest that the need for a pyloric drainage procedure may be more related to the size of the gastric conduit, in that larger conduits (whole stomach) are more susceptible to gastric stasis [8].

So, the aim of the current study was to determine the value and role of pyloromyotomy performed with THE (Orringer’s technique) for esophageal cancer in modifying postoperative gastric emptying time.

## Patients and Methods

In this clinical trial study, a prospective analysis of 40 patients, who underwent transhiatal esophagectomy due to esophageal cancer, was performed. Thirty-eight patients had squamous cell carcinoma; two had adenocarcinoma; thirty-one had cancer in the distal one-third; and nine in the middle one-third. All of them underwent THE with Orringer’s Procedure in a period of two years (2007-2009) in Imam Khomeini Hospital Urmia a tertiary referral hospital.

The patients were randomly divided in two groups; Control group (group A) had THE without pyloromytomy (n = 20) and Study group (group B) had THE with pyloromytomy (n = 20).

For randomization, illegible patients were distributed one after the other between the two groups A and B alternatively according to their order of selection e.g. first illegible patient underwent THE without Pyloromyotomy (group A), while the following one underwent THE with pyloromyotomy (group B) and so on.

A single thoracic surgeon with 5 years experience performed all the surgeries. A check list was made including demographic data; history of medication, diabetes, and previous gastric surgery. The pyloromyotomy was performed. The anterosuperior surface of pylorus was selected for pyloromyotomy. As the pylorus was held between the surgeon’s thumb and index finger, a 2 cm longitudinal incision was made. The incision was carried down through the serosa and mucosal coat. The muscle was then separated apart with a hemostat until the mucosa pouts up [11].

Also, informed consents were witnessed from all patients after approval of the study by university ethic and review board.

All the patients had to take chest x-ray and CT SCAN to exclude metastasis.

### Exclusion criteria

Those with diabetes, taking medications, abnormal pyloroduodenal region or with previous gastric surgery were excluded.

All patients underwent gastric scintigraphy 6 month after surgery to determine gastric drainage. Patients on medication such as those enhancing gastric emptying (metoclopramide, erythromycine, domperidone, tegaserod) and opiate analgesic (morphine, codeine, demerol) and anticholinergic antispasmotic were asked to stop medications 2 days prior to scanning.

Patients were not allowed to take tea or coffee or to smoke before the test. They would come one day for liquid and one day for solid phase imaging. The patient would stay in the imaging till 50% of the eaten material would drain from the stomach. They were asked to come early in the morning without taking breakfast, or if they were unable to fast they were asked to refrain from eating for at least 6 hours. Subjects were advised to bring something to spend the time because they had to stay for at least 4 hours under imaging facility. Patients were asked to stay close to facility between imaging.

In the solid phase, technetium-99 m phytate (Tc-99 m) was used in egg. Some butter (10 gm) was added and included in 50 gm bread and was taken in sandwich. The meal was prepared by pouring liquid egg (2 large liquid eggs white) into bowl and mixed in 1 m Ci Tc-99 m phytate, and cooked for 3-5 minutes in non-stick frying pan. A sandwich was made by the cooked egg. Patients were instructed to take the meal in maximum 10 minutes.

In the liquid phase, a glass of orange juice, mixed with 1 m Ci Tc-99 m DTPA was served.

Patient would lay supine on the imaging table, posterior images were acquired 60 frame with one minute duration one minute each for solid phase and 30 frame with 1 minute duration for liquid phase were obtained (256’256 matrix), with a 20% widow centered on the Tc-99 m photopeak (140 keV). Static images were acquired 2, and 4 hour after meal was ingested, if the drainage was incomplete then we evaluate images with quantitative analyses with drawing of region of interest (ROI) at second and fourth hours.

For liquid emptying, a half-emptying time (time required for the emptying of half the meal) and a best-fit exponential emptying rate (T 1/2) were calculated. Emptying of less than 90% at 4 h was considered delayed. The normal gastric emptying is considered when 50% of activity in stomach at time zero; should empty by 60 ± 30 minutes [12].

### Statistical analysis

We used T-Student’s test for analysis and the P-value of less than 0.05 was considered significant.

## Results

The 40 patients enrolled in the study were divided in two groups. Thirty-eight patients had squamous cell carcinoma; two had adenocarcinoma; thirty-one had cancer in the distal one-third; and nine in the middle one-third. In group A 17 patients had squamous cell carcinoma (14 upper third and 3 in middle one third) while 2 had Adenocarcinoma (all three is lower third). In group B 19 patients had squamous cell carcinoma (15 in upper third and 2 in lower third). None of the patients vomited the meal. In the solid phase, the gastric half emptying time (T50) showed mean ± SD of 141.15 ± 48.42 minutes in the study group B, and 139.40 ± 94.16 minutes in the control group A (P = 0.941) which is not significant. In the liquid phase, the mean gastric half emptying time (T50) showed mean ± SD of 62.85 ± 59.35 minutes in the study group B, and 74.5 ± 56.71 minutes in the control group A (P = 0.529) which is also not significant.

## Discussion

A gastric conduit is usually used as esophageal replacement after vagotomized THE for esophagus cancer. The GE may be impaired after this operation [13, 14], so some esophageal surgeons routinely add pyloric drainage procedures (pyloroplasty or pyloromyotomy).

Previous literatures [15, 16] recommended the use of pyloroplasty on every patient to prevent the potentially lethal effects of gastric stasis in the early postoperative period following retrosternal reconstruction of the oesophagus, especially if the whole stomach is used for esophageal substitution.

Pyloromyotomy is the pyloric drainage procedure used in the present study. Pylorus is fan-shaped specialized circular muscle fibers with: (a) distal sphincter loop = right canalis loop (corresponds to radiologic pyloric sphincter); (b) proximal distal sphincter loop = left canalis loop, 2 cm proximal to distal sphincteric loop, on greater curvature (seen during complete relaxation). The fibers of both sphincters converge on the lesser curvature side to form a muscular prominence (torus). Prolapse of mucosa between sphincteric loops produces a niche simulating ulcer. Pyloric canal = 5 - 10 mm long, wall thickness of 4 - 8 mm. with concentric indentation of the base of the duodenal bulb [17].

The results of the current study showed that after 6 months of performing THE in 40 patients with esophagus cancer, no significant difference in the GE between the control group (without pyloromyotomy) and the study groups (with pyloromyotomy). This appeared in both liquid and solid phases. These results coincide with previous literatures utilized pyloroplasty as a gastric drainage procedure [6, 8].

In agreement with the results of the current study, Lanuti et al [5] reported that pyloromyotomy does not reduce the incidence of symptomatic delayed GE after esophagectomy, and the post-operative GOO can be effectively managed with endoscopic pyloric dilatation. Urschel et al [4] showed a non-significant trend favoring pyloric drainage (in general) for the late outcomes of gastric emptying, nutritional status, and obstructive foregut symptoms.

Palmes et al [18] recommended omission of pyloric drainage procedures after THE with gastric conduit reconstruction because it did not improve GE, but significantly favored postoperative biliary reflux esophagitis compared to those without pyloric drainage. Law et al [17] documented that 6 months after THE, the median half-life for GE was significantly more in the control group than in the pyloromyotomy group. However, long-term follow-up up to 5 years did not reveal significant differences between the two groups in the type and quantity of food consumed. A study, evaluating GE 6-8 weeks after the operation, concluded that all pylorus drainage procedures behave in much the same way; and patients may develop some problems which disappear in due course after proper adjustments in both posture and diet [19].

As a result of the present study, absence of significant difference in GE between control and study groups after 6 months of the surgery might be explained by the gradual return of the tone to the vagally denervated stomach used as an esophageal substitute after a period of time which varies in different patients [9, 20]. The improvement of symptoms after a period of time might be attributed to that the stomach conduit which might act as contractile organ, even generating complete migrating motor complexes. This can results in readjustment of the local active motor mechanism residing in the terminal antrum or pylorus [21]. Similar results were reported following truncal vagotomy and pyloroplasty [22].

We suggest that the actual vagal denervation of the stomach starts gradually preoperatively due to local infiltration of the vagal trunks by the tumor. This might give time to the local antropyloric neuromuscular mechanisms to return to normal after a period of time. Moreover, abrupt surgical truncal vagotomy might explain the initial GOO symptoms.

The pyloric region is supplied by pyloric arteries which are rami of the right gastric and right gastroepiploic arteries. They pierce the duodenum distal to the sphincter around its entire circumference. They pass through the muscular layer to the submucosa where they divide into two or three rami, which turn back into the pyloric canal beneath the mucosa and run to the end of the pyloric antrum. They supply the entire mucosa of the pyloric canal. Branches of these pyloric submucosal arteries may anastomose close to their origin with the duodenal submucosal arteries. Their terminal rami also anastomose with gastric arteries from the prepyloric antrum. The pyloric sphincter is supplied by the gastric and pyloric arteries via rami that leave their parent vessels in the subserosal and submucosal levels to penetrate the sphincter [23].

The results of Marchand [6] recommended using the easier pyloric stretching method rather than pyloroplasty which interferes with the important collateral vessels in the pyloric region, and they may impede mobilization of the stomach by shortening or anchoring the gastric outlet. Like pyloroplasty, pyloromyotomy might also have the same effects on the pyloric collateral vessels which might delay the gradual readjustment and return of the terminal antrum or pylorus to normal.

### Conclusion

We recommend that, in patients with normal pyloroduodenal regions (absent or minimal pyloric hypertrophy, or fibrosis), routine pyloromyotomy might not be performed along with THE, as no significant use of its validity after 6 months of the surgery. However, patients with persistent postoperative gastric outlet obstruction symptoms might be followed by delayed endoscopic pyloric dilatation or internal pyloric stretching.
